# Common 4977 bp deletion and novel alterations in mitochondrial DNA in Vietnamese patients with breast cancer

**DOI:** 10.1186/s40064-015-0843-8

**Published:** 2015-02-03

**Authors:** Jan Dimberg, Thai Trinh Hong, Linh Tu Thi Nguyen, Marita Skarstedt, Sture Löfgren, Andreas Matussek

**Affiliations:** Department of Natural Science and Biomedicine, University College of Health Sciences, Jönköping, Sweden; Key Laboratory of Enzyme and Protein Technology, Department of Biology, College of Science, Vietnam National University, Hanoi, Vietnam; Departments of Clinical Microbiology, Ryhov County Hospital, Jönköping, Sweden; Departments of Laboratory Services, Ryhov County Hospital, SE-551 85 Jönköping, Sweden

**Keywords:** Breast cancer, Mitochondrial DNA mutation, mtDNA deletion

## Abstract

Mitochondrial DNA (mtDNA) has been proposed to be involved in carcinogenesis and ageing. The mtDNA 4977 bp deletion is one of the most frequently observed mtDNA mutations in human tissues and may play a role in breast cancer (BC). The aim of this study was to investigate the frequency of mtDNA 4977 bp deletion in BC tissue and its association with clinical factors.

We determined the presence of the 4977 bp common deletion in cancer and normal paired tissue samples from 106 Vietnamese patients with BC by sequencing PCR products.

The mtDNA 4977 bp deletion was significantly more frequent in normal tissue in comparison with paired cancer tissue. Moreover, the incidence of the 4977 bp deletion in BC tissue was significantly higher in patients with estrogen receptor (ER) positive as compared with ER negative BC tissue. Preliminary results showed, in cancerous tissue, a significantly higher incidence of novel deletions in the group of patients with lymph node metastasis in comparison with the patients with no lymph node metastasis.

We have found 4977 bp deletion in mtDNA to be a common event in BC and with special reference to ER positive BC. In addition, the novel deletions were shown to be related to lymph node metastasis. Our finding may provide complementary information in prediction of clinical outcome including metastasis, recurrence and survival of patients with BC.

## Introduction

The incidence of different cancers have increased both in developed and in developing countries (Jemal et al. 2011). Breast cancer (BC) is one of the most common cancers affecting women worldwide and the incidence is rapidly rising in Asian countries. In Vietnam, the incidence rate is 12 to 27per 100 000 (Anh & Duc 2002; Le et al. 2002) while the incidence for women living in Western countries is about 80 to 100 per 100 000 (Jemal et al. 2011).

The development of BC involves a progression through intermediate states and processes leading to evolution to carcinoma *in situ*, invasive carcinoma and metastasis. Mutations in nuclear genes such as tumor-suppressor genes and oncogenes, but also environmental exposures contribute to the development of BC (McPherson et al. 2000; Polyak 2007; Schwartz et al. 2008). For example high penetrance genes as *BRCA1*, *BRCA2, PTEN* and *TP53* are responsible for the hereditary BC syndromes (Polyak 2007; Schwartz et al. 2008).

It is necessary to identify molecular markers to predict the progression, metastasis, recurrence and survival in BC. Hormone receptors status is used for identifying a high-risk phenotype and to select suitable regime for treatment (Banin Hirata et al. 2014). Other tumor markers suggested useful in diagnostic procedures and for prognosis in BC are expression of chemokines, chemokine receptors and growth factors (Banin Hirata et al. 2014).

Alongside the nuclear genome, the human cell contains hundreds to several thousand copies of the 16 569 base pair circular mitochondrial DNA (mtDNA) including 37 genes (Birch-Machin 2006; Penta et al. 2001). Within cells the mtDNA has the capacity to form a mixture of both wild-type and mutant mtDNA genotypes in a state called heteroplasmy (Birch-Machin 2006; Penta et al. 2001).

mtDNA has been proposed to be involved in carcinogenesis and ageing (Birch-Machin 2006; Penta et al. 2001) and somatic mtDNA mutations have been reported in various types of cancer, including BC (Penta et al. 2001; Chen et al. 2011; Eshaghian et al. 2006; Larman et al. 2012; Yadav & Chandra 2013; Ye et al. 2008). The main reason for its involvement in carcinogenesis is probably that mtDNA has a high susceptibility to undergo mutations due to its lack of histones, limited repair mechanisms and a high rate of generation of reactive oxygen species (Birch-Machin 2006; Penta et al. 2001). The mitochondrial 4977 bp deletion, also known as the common deletion, is one of the most frequently observed mtDNA mutations and has been associated with different cancers (Chen et al. 2011; Eshaghian et al. 2006; Ye et al. 2008; Abnet et al. 2004; Dani et al. 2003). The deletion occurs between nucleotides 8470 and 13 447 and spans five tRNA genes and seven genes encoding subunits of cytochrome *c* oxidase, ATPases and complex I (Chen et al. 2011; Ye et al. 2008). Moreover, the deletion has a 13 bp direct repeat flanking the 5′- and 3′-end breakpoints at nucleotide position (np) 8470/8482 and np 13 447/13 459, respectively (Chen et al. 2011; Ye et al. 2008).

In this study, we determined the frequency of the 4977 bp deletion in BC and corresponding non-cancerous breast tissue samples from 106 Vietnamese patients with BC.

## Materials and methods

### Patients and tissue specimens

This study comprised of 106 consecutive female patients with BC, from northern Vietnam. Tissue specimens were collected when the patients underwent surgical resections at the National Cancer Hospital, Tam Hiep, Hanoi, Vietnam. The mean age of the patients were 52 years (range 24-89 years). Clinicopathological characteristics from the patients were received from surgical and pathological records. Tumor tissue and adjacent normal tissue (about 5 cm from the tumor) from each patient were excised and immediately frozen at 80°C until further analysis.

Clinical and clinicopathologic classification and staging were determined according to the American Joint Committee on Cancer (AJCC) criteria. The tumors (invasive ductal carcinoma) were classified according to TNM staging system and the distribution was: T1N0M0 (n = 8), T2N0M0 (n = 42), T3N0M0 (n = 5), T1N1M0 (n = 2), T1N2M0 (n = 2), T2N1M0 (n = 28), T2N2M0 (n = 3), T3N1M0 (n = 7), T3N2M0 (n = 1), T4N1M0 (6) and T2N1M1 (n = 2).

Tumor grade of 79 patients was known: well differentiated (n = 6), moderately differentiated (n = 56) and poorly differentiated (n = 17). In 24 cases information regarding positive and negative expression of estrogen receptor (ER), progesterone receptor (PR) and human epidermal growth factor receptor 2 (HER2) in tumor tissue, was available. ER + (n = 12), PR + (n = 5) and HER2 + (n = 19). The study was approved by the local Ethics Committee at the Vietnam National University, Hanoi, Vietnam (2422/QD-KHCN) and all patients gave their consent to participate in the study.

### PCR assay

DNA was isolated from all BCs and paired normal tissues by QIAamp DNA Mini kit (Qiagen, Hilden, Germany). To screen for the mitochondrial 4977 deletion, a nested PCR was developed to detect low levels of the deletion. Two pairs of PCR primers were designed for the first amplicon of 496 bp and the second amplicon of 381 bp (Table 1). For the first amplicon, the primers were designed to be distant enough to detect only mtDNAs containing deletions. To assess the presence of mtDNA and to detect heteroplasmy/homoplasmy regarding 4977 deletion, PCR primers were designed in the region of the genes NADH dehydrogenase 1 (*ND1*) and *ND3* resulting in products of 433 bp and 246 bp, respectively (Table 1).

**Table 1:** **Primer sequences and product sizes for mtDNA 4977 bp deletion analysis in this study**

**Primer**	**Primer sequence**	**Position**	**Product**	**Note**
mtDNA-forward	5′-GACGCCATAAAACTCTTCAC-3′	3457-3476	433 bp	ND1-region
mtDNA-reverse	5′-GGTTGGTCTCTGCTAGTGTG-3′	3889-3870		
4977-1forward	5′-TCAATGCTCGAAATCTGTGG-3′	8167-8187	496 bp	First PCR
4977-1reverse	5′-GTTGACCTGTTAGGGTGAGAAG-3′	13639-13618		
4977-2forward	5′-ACAGTTTCATGCCCATCGTC-3′	8196-8215	381 bp	Second PCR
4977-2reverse	5′-GCGTTTGTGTATGATATGTTTGC-3′	13553-13531		
10398-forward	5′-CCTGCCACTAATAGTTATGTC-3′	10307-10327	246 bp	ND3-region
10398-reverse	5′-GATATGAGGTGTGAGCGATA-3′	10552-10533		

Except for the second PCR run for 4977 deletion, DNA was amplified in a total volume of 12.5 μl containing 0.2 μM of each primer (TIB Molbiol, Berlin, Germany), 1.8 mM MgCl_2_, 200 μM of each deoxynucleotide triphosphate, 0.04 units *Taq* DNA polymerase and reaction buffer [20 mM Tris-HCl (pH 8.3), 20 mM KCl, 5 mM (NH_4_)_2_SO_4_] (Fermentas, Burlington, Canada). Amplification was done with an initial denaturation at 95°C for 4 min followed by 35 cycles at 92°C for 30 s (denaturation), 54°C for 30 s (annealing), 72°C for 45 s (extension) and final elongation at 72°C for 10 min. For the second PCR run regarding the 4977 deletion, the conditions were the same as above except that an annealing temperature of 60°C and a total number of 32 cycles was used. The amplified PCR products were visualized by UV-illumination on 2% agarose gel containing Gel Red (Biotium, Inc., Hayward, CA). The band reflecting the 4977 common deletion and all the other bands that were obtained at different levels on the gel were purified with Gel Extraction kits (Qiagen, Hilden, Germany), followed by commercial sequencing (GATC Biotech, Köln, Germany).

### Statistical analysis

Differences in the rate of mtDNA deletions were analyzed using the Chi-square test. Statistical analyses were performed using SPSS for Windows computer package (IBM SPSS Statistics, 2012, version 19; SPSS Inc., Chicago, IL). Results were considered significant at *p* < 0.05.

## Results

### Frequency of mtDNA 4977 bp deletion in patients with BC

**Figure 1:**
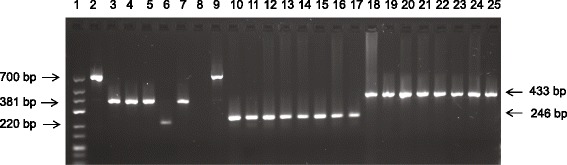
**Agarose gel showing polymerase chain reaction (PCR) products from four breast cancer tissue/normal paired tissue.** Nested PCR (381 bp, lane 2/3, 4/5, 6/7, 8/9); 10398 (246 bp, lane 10/11, 12/13, 14/15, 16/17); mtDNA (433 bp, lane 18/19, 20/21, 22/23, 24/25) and discovered novel deletions (700 bp, lane 2 and 9; 220 bp, lane 6). Lane 1, molecular marker.

All samples showed clear bands with mtDNA and 10398 primers representing 433 bp and 246 bp respectively (Figure 1). In lanes 2, 6 and 9 (Figure 1), three novel deletions were detected (700, 220 and 700 bp, respectively) which were confirmed by sequencing. For the 4977 bp deletion, represented by bands 381 bp, we defined two types of signals by nested PCR: negative and positive clear band (Table 2). The deletion was detected in 68.8% (73/106) of cancerous tissues and 84.0% (89/106) of normal paired tissues (Table 2) (*p* < 0.01).

**Table 2:** **Mitochondrial DNA 4977-bp deletion in Vietnamese patients with breast cancer**

		**Prevalence of deletion (n)**	
**Parameters**	**No. of cases**	**Negative**	**Positive**
Cancer tissue	106	33	73
Normal paired tissue	106	17	89
Stage*			
T1N0M0	8	2	6
T2N0M0	42	12	30
T3N0M0	5	2	3
T1N1M0	2	1	1
T1N2M0	2	2	0
T2N1M0	28	6	22
T2N2M0	3	2	1
T3N1M0	7	2	5
T3N2M0	1	0	1
T4N1M0	6	3	3
T2N1M1	2	1	1

*Cancer tissue.

With regard to disease stage, the patients were divided into two sub-groups, one with no metastasis to lymph node or other organs (T1-3, N0, M0) and one with spread (T1-4, N1-3, M0-1). However, no significant difference was seen with respect to the frequency of 4977 bp deletion. Nor were tumor grade or age associated with the 4977 bp deletion (data not shown).

We found a significantly (*p* < 0.01) higher rate of the 4977 bp deletion in patients with ER+, 91.2% (11/12) compared with ER−, 41.2% (5/12). Neither PR nor HER2 showed statistically significant correlation to the presence of 4977 bp deletion.

### Detection of novel mtDNA deletions

After nested PCR, we detected different bands in addition to the 381 bp which represents the 4977 bp deletion. The bands that were both larger and smaller than 381 bp were purified, sequenced and the corresponding deletions were analyzed using the program BLASTn (Altschul et al. 1990). The deletions were checked against the MITOMAP database (MITOMAP 2013) and other possible reference sources, with the consequence that we characterize our findings as novel deletions. Tables 3 and 4 summarize the novel deletions in tumor and normal tissue with information about breakpoints, deletion size, repeat location and type, respectively. We found 36 novel deletions in the tumor tissue distributed among 33 patients and 30 novel deletions in the normal tissue spread over 26 patients.

**Table 3:** **Novel mtDNA deletion (n = 36) detected in breast cancer tissue**

**Patient code**	**Deletion junction (nt:nt)**	**Deletion size (bp)**	**Repeat location (nt)**	**Repeat type**
8	8712:13256	4543	8709-8711/13256-13258	I, 3/3
10	8318:13500	5181	-	NR
11	8249:12960	4710	-	NR
20	8228:13479	5250	8228/13478	D, 1/1
26	8329:13411	5081	8330-8333/13409-13412	I, 4/4
28	8300:13448	5147	-	NR
30	8439:13080	4640	8435-8439/13074-13079	D, 5/6
31	8241:13278	5036	8241/13277	D, 1/1
32	8405:13165	4759	8404-8405/13163-13164	D, 2/2
33	8553:13206	4652	8552-8553/13206-13207	I, 2/2
33	8338:12588	4249	8333-8338/12582-12587	D, 5/6
38	8271:13358	5086	8271/13357	D, 1/1
39	8532:13397	4864	8526-8532/13390-13396	D, 7/7
41	8586:13457	4870	8582-8586/13452-13456	D, 4/5
44	8282:13488	5205	8279-8282/13484-13487	D, 4/4
44	8309:13474	5164	8310-8315/13474-13479	D, 6/6
52	8256:13412	5155	-	NR
53	8436:13528	5091	8430-8436/13520-13527	D, 5/7
55	8223:13415	5191	-	NR
56	8319:13498	5178	8320-8321/13498-13499	I, 2/2
60	8272:12908	4635	8272/12907	D, 1/1
61	8474:13525	5050	8463-8474/13514-13524	D, 10/12
68	8273:13138	4864	-	NR
69	8227:13422	5194	8227-8228/13420-13421	I, 2/2
70	8448:13499	5050	-	NR
73	8216:13473	5256	8216/13472	D, 1/1
76	8262:13415	5152	8260-8262/13412-13414	D, 2/3
77	8354:13411	5056	-	NR
79	8252:13490	5237	-	NR
86	8324:13491	5166	8310-8324/13474-13490	D, 13/17
90	8282:13488	5205	8279-8282/13484-13487	D, 4/4
91	8296:13372	5076	8294-8296/13370-13372	D, 3/3
99	8222:13440	5217	8222/13439	D, 1/1
101	8443:13496	5052	8441-8443/13492-13495	D, 3/4
102	8369:12552	4182	8370-8379/12551-12559	I, 9/10
102	8505:13405	4899	8503-8507/1340-1344	I, 5/5

D, direct repeat; NR, no repeat; nt, nucleotide; I, indirect repeat.

**Table 4:** **Novel mtDNA deletion (n = 30) detected in breast normal tissue**

**Patient code**	**Deletion junction (nt:nt)**	**Deletion size (bp)**	**Repeat location (nt)**	**Repeat type**
4	8251:13414	5162	8244-8250/13409-13415	D, 7/7
6	8257:13447	5189	8257/13446	D, 1/1
8	8226:13459	5232	8225-8227/13459-13461	D, 3/3
9	8326:13480	5153	8327-8328/13479-13480	D, 2/2
11	8332:13210	4877	-	NR
19	8313:13522	5208	8314-8316/13521-13523	D, 3/3
19	8300:13206	4905	8299-8300/13204-13205	D, 2/2
20	8263:13461	5197	8259-8263/13457-13461	I, 5/5
20	8231:13328	5096	8228-8231/13328-13332	D, 4/5
24	8564:13334	4769	8560-8564/13328-13332	D, 5/5
28	8305:13533	5227	8304-8305/13531-13532	D, 2/2
32	8256:13313	5056	8254-8257/13309-13312	I, 4/4
38	8435:13474	5038	8434-8435/13472-13473	D, 2/2
41	8396:13466	5069	8395-8396/13464-13465	D, 2/2
43	8299:13463	5163	8294-8299/13457-13462	D, 5/6
50	8234:13286	5051	8231-8234/13283-13285	D, 3/4
52	8297:13428	5130	8295-8297/13425-13427	D, 2/3
52	88801:13462	4660	8787-8801/13448-13461	D, 13/15
59	8355:13440	5084	8343-8355/13428-13439	D, 11/13
61	8216:13396	5179	-	NR
65	8425:13297	4871	8421-8425/13291-13296	I, 5/6
67	8362:13465	5102	8363-8364/13465-13466	I, 2/2
79	8492:13529	5036	8491-8492/13527-13528	D, 2/2
79	8215:13117	4901	8214-8215/13115-13116	D, 2/2
88	9160:12966	3805	9149-9160/12954-12965	D, 12/12
92	8556:13170	4613	8553-8556/13166-13169	D, 3/4
101	8349:13421	5071	8348-8349/13419-13420	D, 2/2
103	8312:13467	5154	8313/13466	D, 1/1
106	8259:12994	4734	-	NR
107	8534:13399	4864	8526-8534/13390-13398	D, 8/9

D, direct repeat; NR, no repeat; nt, nucleotide; I, indirect repeat.

A number of patients with at least one novel deletion in the cancerous tissue were 12 with no involved lymph nodes (N0) and in 21 with involved lymph nodes (N1-2). Moreover, we observed, in cancerous tissue, a significantly (*p* < 0.05) higher rate, 41.2% (21/51), of the novel deletions in the group of patients defined as N1-2 in comparison with 21.8% (12/55), in the group defined as N0. However, this result is not consistent with good statistical power which has a value around 0.6. There were no associations between the novel deletions and other clinical characteristics and no associations in the normal tissue (data not shown).

### Observed novel mtDNA single nucleotide variants

Fifteen novel mtDNA single nucleotide variants were identified in the region sequenced and resident in the novel deletions reported here (Table 5). These were not linked to any clinical parameter available in this study (data not shown).

**Table 5:** **Novel mtDNA single nucleotide variants detected in breast cancer and normal tissue**

**Sample no.**	**Tissue**	**Variant**
10	Cancer	T13543A
19	Normal	T13386A
20	Normal	A13395G
24	Normal	G13414A
43	Normal	T13460C
52	Normal	G8790C
59	Normal	C8349T
61	Cancer	C8472A, A13519C
68	Cancer	A13395G
77	Cancer	C8270T, C13503T
86	Cancer	G13480T, T8317G
104	Cancer	T13488C

## Discussion

The mitochondrial 4977 bp deletion has been found in tissues from several tumor types and adjacent normal tissues (Penta et al. 2001; Chen et al. 2011; Ye et al. 2008; Abnet et al. 2004; Dai et al. 2006). Recently, reduced mitochondrial mutagenesis in colorectal cancer has been shown, as well as a higher frequency of mtDNA mutagenesis, which may prevent colorectal cancer (Ericson et al. 2012). In the present study, the mtDNA 4977 bp deletion was found at a significantly higher frequency in normal tissue in comparison with paired cancer tissue in Vietnamese BC patients. We also observed a pervading heteroplasmy in the tissues. Our results are consistent with a previous study showing decreased proportions of the mtDNA 4977 bp deletion in various cancer types compared with adjacent normal tissue, such as breast (Ye et al. 2008), lung (Dai et al. 2006), gastric (Wu et al. 2005) and colorectal cancer (Dimberg et al. 2014). One explanation of this phenomenon might be a dilution of the mtDNA 4977 bp deletion in tumor tissue as a result of clonal expansion during cancer progression or that cells harbouring this deletion are eliminated by apoptosis (Wu et al. 2005). Moreover, the mtDNA 4977 bp deletion might confer a metabolic disadvantage to proliferating cells and thus is selected out in the highly proliferative tumor tissue (Wu et al. 2005).

Testing the tumor for hormonal receptors is a standard part of a BC diagnosis. In general BC with positive hormonal receptor status tends to be more aggressive and fast growing. Moreover, the receptor status predicts the treatment response and thus will influence the treatment regimen (Goldhirsch et al. 2009). In the present study, we found that the incidence of the 4977 bp deletion in BC tissue is significantly higher in the patients with ER positive as compared with ER negative patients. It has been reported that p53 plays a role in the maintenance of mtDNA integrity by controlling replication and repair through interaction with DNA pol gamma (Achanta et al. 2005). A study demonstrated that ER binds to p53 on the p53 target gene and represses p53 mediated transcriptional activation (Konduri et al. 2010) and may thus explain that 4977 bp deletion seems to be more prevalent among ER positive patients.

In addition to the 4977 bp deletion, we discovered novel large scale deletions, 36 in cancerous and 30 in normal tissue. Moreover, 15 novel mtDNA single nucleotide variants were identified within the region sequenced and resident in the novel deletions reported here.

Interestingly, we observed, in cancerous tissue, a significantly higher incidence of the novel deletions in the group of patients with lymph node metastasis in comparison with the patients with no lymph node metastasis. However, this result is preliminary because of insufficient number of patients. It is possible that our novel deletions are involved in the mediation of tumor progression. However, our finding does not provide answers as to whether mtDNA alterations are contributing factors to carcinogenesis or whether they simply arise as part of secondary effects in cancer progression. Whether our detected novel deletions have an impact on cancer development or not requires further investigation. Studies have shown that a reduced mtDNA content is associated with higher histological grade in BC (Yadav & Chandra 2013) while other studies failed to demonstrate any correlation with tumor grade or metastasis (Yadav & Chandra 2013; Mambo et al. 2005). In the future, it would be of interest to investigate this type of correlation in our group with increased number of patients.

To our knowledge, this is the first time that mtDNA alteration in BC tissue and paired normal tissue has been analyzed in Vietnamese patients. We have focused on identification of the 4977 bp deletion but also on characterization of novel mutations. The results about the novel mutations must be confirmed by expanding the investigation. Studies using increased sample size are required to determine the clinicopathologic role of the sequence variation of mtDNA in BC. Our finding may provide complementary information in additional studies to define the importance of the mtDNA deletions found in prediction of clinical outcome including metastasis, recurrence and survival of patients with BC.
